# Simulation of Inclusion Particle Motion Behavior under Interfacial Tension in Continuous Casting Mold

**DOI:** 10.3390/ma15217458

**Published:** 2022-10-24

**Authors:** Md Irfanul Haque Siddiqui, Ayidh Albaqami, Latif Arifudin, Khalid Alluhydan, Ibrahim Abdullah Alnaser

**Affiliations:** 1 Department of Mechanical Engineering, King Saud University, Riyadh 11451, Saudi Arabia; 2 Center of Excellence for Research in Engineering Material (CEREM), King Saud University, Riyadh 11451, Saudi Arabia

**Keywords:** alumina inclusion, interfacial tension, steelmaking, continuous casting mold, surfactant, sulfur, simulation

## Abstract

Inclusions entrapped by the solidifying front during continuous casting adversely affect the properties of the final steel products. In this study, we investigated the effect of the interfacial tension due to surfactant concentration, particularly sulfur, on alumina inclusion motion behavior during molten steel solidification in a continuous casting mold. A two-dimensional numerical model was developed in Ansys Fluent software to simulate the inclusion motion in a continuous casting mold. Further, the impacts of different values of the alumina inclusion diameter, sulfur concentration, and melt temperature were studied to understand the inclusion motion behavior. The inclusion diameter affected the inclusion distribution throughout the domain. The alumina inclusion entrapment percentage varied in the case of sulfur mixing (using an empirical relationship for modeling). It was found that the removal percentage varied according to the sulfur concentration. The addition of sulfur at concentrations from 10 ppm to 70 ppm resulted in a 4% increase in the removal of alumina inclusions (trapped in the solidifying shell), except for the 100-ppm case. Smaller-sized inclusion particles had a 25% higher chance of entrapment at the top level of the mold. Under the effect of a higher surface tension gradient between inclusions and the melt, the predicted findings show that inclusions were vulnerable to engulfment by the solidification front.

## 1. Introduction

Steel’s mechanical properties and chemical composition are continually changing, and worries about cost, energy, and the environment in the manufacturing process are becoming increasingly relevant. To fulfill demand, steel’s strength, ductility, durability, and corrosion resistance have all increased over time. A variety of processes, strongly influenced by the equipment, modify the final product’s shape, appearance, and properties [1]. Due to its superior features, steel has been widely used for various applications since it was commercialized at the beginning of the 1900s. The need for clean steel is an important aspect for steel producers in the context of greenhouse emissions, competition, customer requirements, and sustainability. It has been reported widely that nonmetallic inclusions significantly influence the properties of steel products. The mold remains the last stage in the continuous casting process, where the proper implementation of processes can lead to significant inclusion removal. To properly control nonmetallic inclusions during the continuous casting process, it is of the utmost importance to understand the inclusion motion behavior in the continuous casting mold [2,3,4].

### 1.1. Inclusions in the Continuous Casting Mold

There are two kinds of nonmetallic inclusions in steel, each with its unique mode of formation. Indigenous oxide inclusions, which form when the steel melt is deoxidized, are one kind. The bulk of these oxides is removed from the melt during refining and degassing in the ladle, but some nonmetallic oxide inclusions remain. When the deoxidized steel melt is reoxidized by air or entrained slag is introduced into the melt during the melt transfer from the ladle to the mold, exogenous inclusions occur. Exogenous inclusions are generally much larger than native inclusions and hence more harmful. Inclusions cause problems during the casting, rolling, and heat treatment processes, and they may cause the steel to break during use. The size and nature of nonmetallic inclusions that decrease steel properties are not constant but rather fluctuate according to the application [5,6,7,8,9].

Typically, in the secondary steelmaking process, oxygen impurities are removed by aluminum deoxidation. However, alumina inclusions are formed during the deoxidation process. The steel components and structure require control of alumina inclusions to ensure their design performance. Inadequate removal of alumina inclusions during the steelmaking process can induce micro-surface cracks during continuous casting and hot rolling [10,11]. Generally, solid nonmetallic inclusions are not well-wetted with liquid steel. Inclusions distributed in liquid steel are sometimes engulfed in the solidification interface in the mold and become defects in steel products. Inclusions entrapped by the solidifying front during continuous casting will adversely affect the properties of the final steel products [1,12,13,14]. Therefore, the behavior of inclusions at the interface should be investigated in detail.

### 1.2. Inclusion Motion Characteristics

**Figure 1:**
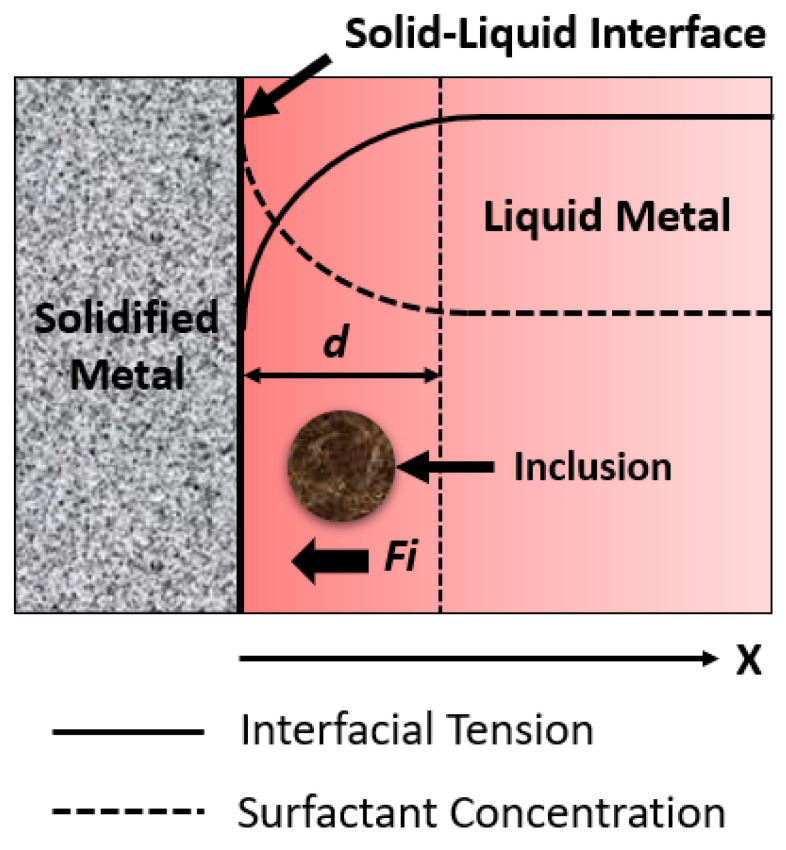
The effect of Marangoni force on an inclusion near the solidifying front (d = distance between solidifying boundary and saturation-level interfacial tension; Fi = interfacial tension).

The interfacial tension, which is also referred to as the pushing and engulfment phenomenon, has an impact on the distribution of inclusion particles trapped in the solidifying slab of the continuous casting mold. The interfacial characteristics are connected to the pushing and engulfment of inclusion particles [15,16,17]. Particles move from one position with higher interfacial tension to another position with lower interfacial tension when there is a gradient of interfacial tension around a gas bubble or a small liquid inclusion. The Marangoni force’s impact on inclusions at the solid–liquid border is seen in Figure 1. As is the case for pure metals, surface tension typically decreases as temperature rises. The presence of a surface-active element in a metal is not necessarily a given, though. To regulate Marangoni convection in the molten metal and the subsequent solidification in the mold, the concentration of minor elements in steels is important [18]. Some researchers have used bubbles to conduct extensive research on this phenomenon in water model experiments. Mukai et al. [15,16,17] reported that the pushing and engulfment of small bubbles occur in the solid–liquid interface due to the presence of a gradient of interfacial tension in the boundary layer. Further, Shibata et al. [19] studied the inclusion/bubble motion behavior at the solid–liquid interface of the solidifying metal surface. Some researchers emphasize that the Marangoni effect will affect the entire process of microgravity experiments due to temperature gradients or concentration gradients of surfactant elements [18,20]. Yin and Emi [20] reported that Marangoni flow was vigorously strong in molten steel, even with low concentrations of oxygen and sulfur. J. Jeong et al. (2020) [11] carried out comprehensive experimental work to find the correlation of interfacial tension between alumina inclusions and molten automotive steel. They studied the effect of surfactant concentration (sulfur) and temperature on interfacial tension.

### 1.3. Background of the Research

As a structural material, steel has benefits such as strength, ductility, and durability. Durability relates to resistance to wear, fatigue, hydrogen-induced cracking, and stress corrosion cracking, whereas ductility is exemplified by properties such as deep drawability, cold formability, and low-temperature toughness. Large nonmetallic impurities significantly reduce steel’s ductility and durability. These inclusions are known as “dirty” inclusions, and they are seen in steel. The increasing need for enhanced ductility and durability in demanding applications can only be met by “clean” steels, which have a smaller number of evenly distributed, small-size impurities.

Oxides, sulfides, nitrides, carbides, and their compounds or composites are examples of nonmetallic inclusions. During typical cooling of steel below the solidus temperature, sulfides, carbides, and nitrides precipitate. To improve steel qualities, small particles of certain oxide inclusions, sulfides, carbides, and nitrides have been used to manipulate the microstructure. Most big oxide inclusions and certain sulfide inclusions, on the other hand, occur when the steel is still liquid. They may create errors in casting goods, cause processing problems and failures, lower productivity, impair product quality, and diminish premium yield if they are not eliminated from the steel melt before solidification.

Improvement of the performance of continuous casting processes has been a subject of extensive research in the metallurgical industry. High-speed casting in the continuous casting operation is an essential objective of many of these studies, where the goal is to improve productivity without sacrificing the quality of the casts. In the continuous casting process, the fluid flow phenomenon greatly affects the quality of steel and defects in the final steel product [21]. It is part of a long-term effort to develop and apply comprehensive models of continuous casting and is supported by computational models and experimental measurements.

The particles in the liquid melt can move toward or away from the freezing front during the solidification of a liquid containing scattered second-phase particles; particles close to the freezing front will either be consumed or rejected. During solidification, these two events cause the particle distribution to change. The molten steel flows in the continuous casting process include several interacting phenomena that interact with each other in a complex form, such as heat transfer, solidification, multiphase turbulent flow, clogging, electromagnetic effects, complex interfacial behavior, particle entrapment, thermal-mechanical distortion, stress, cracks, segregation, and microstructure formation [22,23,24]. In the continuous casting mold, studies on fundamental phenomena, such as temperature, solidified shell growth, turbulent fluid flow along with multiphase physical processes, electromagnetic effects and particle transport, microstructure and grain structure, thermal properties, distortion, and stress, have all made significant strides toward accurately predicting these factors by computational models. However, the accurate prediction of the inclusion motion behavior near the solidifying boundary layer in response to the surfactant concentration needs much further study [25]. Mukai and Masafumi (2003) [15] studied the motion of inclusion particles at the solidifying boundary layer. They reported that the movement of inclusion particle motion due to the surfactant gradient is inextricably linked to some of the phenomena in the casting process, such as bubble- or inclusion-related defects in steel. This effect may be noticed due to the presence of a surface tension gradient around the solidifying boundary induced by the surfactant concentration. Yin et al. (2018) [26] studied inclusion motion and entrapment during full solidification in a curved billet caster. They created a numerical model in three dimensions linking flow, solidification, and inclusion motion. It was noted that the distribution of inclusions inside the mold’s liquid pool is not exactly symmetrical. Additionally, the flow pattern of the molten steel and the inclusions’ buoyancy force have a significant impact on the mobility and trapping of tiny inclusions in the mold.

Z. Liu and B. Li (2018) [27] developed a model to simulate the transient fluid flow, heat transfer, and solidification processes in various vertical-bending continuous casting casters. The enthalpy-porosity approach was used to simulate the heat transfer and solidification of steel in the caster. The vertical length has less effect on the removal ratio of inclusions from the top surface but determines the entrapment positions of inclusions. More inclusions can be carried and captured deep in the mold cavity with a larger vertical length. J. Jeong et al. (2020) [11] predicted the behavior of an alumina inclusion in front of the solid–liquid interface during solidification, and the interfacial tension between SPFH590 microalloyed steel and alumina was experimentally determined. According to the research results, the surface stresses of steel samples decreased as the temperature rose. Additionally, the contact angles of samples containing 11 to 72 ppm sulfur decreased with rising temperature, but they rose for the sample containing 94 ppm sulfur. The estimated interfacial tension values were used to anticipate the behavior of an alumina inclusion in front of the solid–liquid interface in SPFH590 steel. According to estimates, inclusions transitioned from being trapped to being driven away from the solid–liquid interface as the sulfur concentration rose from 5 to 10 ppm.

Using the empirical results established by Ref. [11], Siddiqui et al. (2020) [28] used a novel correlation of the interfacial tension of alumina inclusions in molten steel under different conditions of sulfur concentration to study inclusion motion at the solid–liquid boundary. It was reported that inclusions were prone to engulfment by the solidification front under the influence of higher interfacial tension between the inclusions and the melt.

H. Yin and T. Emi (2003) [20] investigated the direction and velocity of the surface flow of the steel melt in the vicinity of the solid–melt interface. It was found that the solutal Marangoni flow was very strong during the solidification of the steel melt with very low O and S contents. Further, many fine inclusion particles were brought to the free surface near the interface by this flow. Fei et al. (2019) [29] investigated the surface flaws in interstitial-free (IF) steel and carried out quantitative metallographic assessments of near-surface inclusions as well as surface liquid flow detection using the nail-board tipping method. The findings demonstrate that at casting rates of 0.8 and 1.0 m/min, a thin liquid mold flux layer develops, non-uniform argon bubble floating causes the entrainment and subsequent entrapment of the liquid flux, and small inclusion particles of Al_2_O_3_ may also collect at the solidification front.

Q. Wang and Zhang (2016) [30] studied the entrapment and final distribution of inclusions in the solidified shell. Their results indicated the distribution of oxide inclusions along the radial direction. Further, with a larger diameter, inclusions tended to be entrapped toward the center area of the billet. Chen, Ren, and Zhang (2018) [31] investigated the fluid flow, solidification, motion, and entrapment of inclusions in an actual vertical-bending continuous casting (CC) strand. It was reported that the inclusion removal fraction increased significantly with the increase in inclusion diameter, while the percentage of large-size inclusions in the entrapped inclusions was decreased. Kölbl and Harmuth (2019) [32] reported that the quality of steels is mainly influenced by the infiltration of mold slag into the gap between the strand and the mold and the temperature-dependent solidification behavior of the slag. You et al. (2017) [2] reported that the formation of nonmetallic inclusions in the solidification process can essentially influence the properties of steels. They stated that microsegregation and inclusion formation are fundamental to simulating the phenomenon in the solidification process. Wein Chen et al. (2019) [33] carried out work in which a coupled three-dimensional model, the Lagrangian Discrete Phase Model, and a VOF multiphase model were developed to investigate the transient two-phase flow and bubble distribution in continuous casting strands. The influence of the lift force, bubble diameter, and argon flow rate on the transient two-phase flow and bubble distribution were investigated. Soumava Chakraborty et al. (2020) [34] studied the efficiency of the removal of solid alumina inclusions by filtration and the distribution of inclusions in a stainless steel casting. The study documented that inclusion floatation inside the mold cavity plays a role in reducing the inclusion concentration in the casting.

It is noted from the literature review that several techniques have been implemented to prevent inclusions in steel, particularly in a continuous casting mold. It is also evident that the inclusion motion behavior and entrapment in the mold are dependent upon several factors, such as thermo-physical properties and molten steel flow characteristics. Amongst these variables, the surfactant concentration plays an important role in inclusion motion near the solid–liquid boundary layer. The concentration level affects the interfacial tension, and thus, Marangoni forces play an important role in the pushing and engulfment phenomenon. The interfacial tension gradient along the interface between liquid and inclusion particles will propel the particle in the direction of decreasing interfacial tension. It is established in studies in the literature that inclusion particles are driven in the mold by the interfacial tension gradient, especially in front of a solidifying interface and in the vicinity of the interface between liquid steel [35]. Further, it is also evident that minor concentrations of certain surfactants, such as oxygen, nitrogen, and sulfur, play an important role in inducing interfacial tension at the solid–liquid boundary.

In most of the previous studies, inclusion motion has been characterized in terms of fluid flow movement in a continuous casting mold. Very few experimental studies have reported on the inclusion motion behavior under Marangoni forces induced by the surfactant concentration in the continuous casting mold. None of the experimental or numerical research works has reported on inclusion motion using experimental correlations between interfacial tension, surfactant concentration, and temperature. Amongst the few available numerical works, no research has been reported to identify the effect of sulfur concentration on the pushing and engulfment of alumina inclusions near the solidifying front of automotive steel in a continuous casting mold.

In this work, we investigated the inclusion motion behavior in a continuous casting mold using two-dimensional turbulent modeling. The inclusion motion characteristic has been studied with different parameters, such as the input temperature of molten steel in the mold and the size of the inclusion particles. Further, we focused on inclusion motion in the solidifying zone. This research study focused on alumina inclusion motion behavior in molten automotive steel (SPFH590) in a continuous casting mold. Firstly, a full-scale model of the mold was utilized to simulate the melt flow and inclusion flow characteristics depending on some fundamental parameters. Secondly, interfacial correlation and thermo-physical property data from the literature were used for the simulation. In this work, the numerical simulation was simplified by utilizing only important phenomena, such as heat transfer and the interfacial tension distribution in a two-dimensional domain, assuming low to negligible velocity at the solid–liquid interface.

## 2. Numerical Modeling

A computational fluid dynamic-based numerical model was developed by using Ansys Fluent to simulate the molten steel flow and alumina inclusion motion in the continuous casting mold. Table 1 shows the alloying elements of the steel modeled in this study. The simulation model considers the molten steel flow, heat transfer, solidification of molten steel, and inclusion motion. Therefore, a two-dimensional model was used for the simulation. The study was carried out using different parameters, such as the surfactant concentration and the temperature of molten metal. Table 2 and Table 3 show the details of thermo-physical properties for numerical modeling.

The experimental data for the interfacial tension of alumina inclusions and other thermo-physical properties were from the research works of J. Jeong et al. (2020) [10] and Siddiqui et al. [28].

### The Governing Equations

The numerical model was developed to simulate the melt flow and predict the inclusion motion behavior in a continuous casting mold. In the first stage of the research work, we used a full-scale model of the continuous casting mold for the simulation. A transient two-dimensional numerical model was developed using Ansys Fluent (Canonsburg, PA, USA). The inclusion particles were incorporated using the discrete phase modeling method. In addition, the computational fluid dynamics (CFD) model was based on a realizable k-epsilon model and was initially treated as a single-phase model. A major advantage of the realizable k-epsilon model is its ability to accurately predict the spreading rate of both planar and round jets, which is highly related to this work.

In the second stage of the work, we used small geometry to understand the alumina inclusion motion near the solid boundary. In order to investigate the impact of weld parameters and other interfacial phenomena, a two-dimensional multiphase model was built for this example. The governing equations for mass and momentum for the current simulation model, which was created using a mathematical model based on CFD, are as follows:

(1) $$\frac{\partial \rho }{\partial t}+\nabla \cdot \left(\rho \vec{v}\right)=S_{m}$$

(2) $$\frac{\partial }{\partial t}\left(\rho \vec{v}\right)+\nabla \cdot \left(\rho v\vec{v}\right)=-\nabla p+\nabla \cdot \left(\overset{=}{\tau }\right)+\rho \vec{g}+\vec{F}$$

where *p* denotes the static pressure, *g* denotes the gravitational constant, *S_m_* denotes the mass source term, and *F* is the external gravitational force. The velocity field, pressure, and temperature of the fluid in the specified domain are determined using the solutions of the aforementioned Equation contains the stress tensor $\overset{=}{\tau }$ information (3).

(3) $$\overset{=}{\tau }=\mu \left[\left\{\nabla \vec{v}+\nabla {\vec{v}}^{T}\right\}-\frac{2}{3}\nabla \cdot \vec{v}I\right]$$

where μ is the molecular viscosity, *I* is the unit tensor, and the second term on the right-hand side is the effect of volume dilation. The energy equation is expressed in Equation (4):

(4) $$\frac{\partial }{\partial t}\left(\rho E\right)+\nabla \cdot \left\{\vec{v}\left(\rho E+p\right)\right\}=\nabla \cdot \left\{k_{eff}\nabla T-\underset{j}{\sum }h_{j}{\vec{J}}_{j}+\left({\overset{=}{\tau }}_{eff}\cdot \vec{v}\right)\right\}+S_{h}$$

where *k_eff_* is the effective conductivity (*k + k_t_*, where *k_t_* is the turbulent thermal conductivity, defined according to the turbulence model being used), and ${\vec{J}}_{j}$ is the diffusion flux of species *j*. *S_h_* is a volumetric heat source term.

Melt pool (molten metal) solidification was quantified using the enthalpy-porosity approach. The mushy zone, or the liquid portion of molten metal in the range of 0 to 1, was regarded as a porous medium. The liquid fraction of each cell was determined after taking the porosity into account. Additionally, the solidified cells were handled as if they had a porosity percentage of one and were non-porous. As a result, it was assumed that the velocities of fully solidified cells were zero. Additionally, a “pseudo”-porous medium was developed to represent the mushy zone. This indicates that the porosity percentage in the mushy zone ranges from 0 to 1. The sensible enthalpy, *h*, and the latent gas were combined to compute the metal enthalpy in this model:

(5) H=h+ΔH

where

$$h=h_{ref}+\int_{T_{ref}}^{T}C_{p}dT$$

Additionally, *h_ref_* is the reference enthalpy, *T_ref_* is the reference temperature, and *C_p_* is the specific heat at constant pressure.

Further, the liquid fraction, *£*, can be defined as:

£=0 if Temperature (To)<Solidus Temperature(Ts)

£=1 if Temperature (To)>Solidus Temperature(Ts)

$$£=\frac{To-Ts}{Tl-Ts}\;if\;Ts<T<Tl$$

The molten metal’s latent heat content is given as *L*, Δ*H = £*. For both solids and liquids, the latent heat content ranges from 0 to 1. The value of latent heat can be obtained from. Furthermore, the energy equation for solidification/melting problems is written as:

(6) $$\frac{\partial }{\partial t}\left(\rho H\right)+\nabla \cdot \left(\rho \vec{v}H\right)=\nabla \cdot \left(k\nabla T\right)+S$$

where *S* is the source term, and *H* is the enthalpy. The fundamental strategy used to handle melting and solidification is to modify the thermal energy equation by including a phenomenological heat source factor. The domain started with a certain temperature of molten steel at the start of the calculation. The initial circumstances were used to determine the heat source term.

The local mass fraction of molten steel, sulfur material, *Y_i_*, is predicted by the solution of a convection–diffusion equation for the ith species. The conservation formula for all liquid phases is as follows:

(7) $$\frac{\partial }{\partial t}\left(\rho Y_{i}\right)+\nabla \cdot \left(\rho \vec{v}Y_{i}\right)=-\nabla \cdot {\vec{J}}_{j}$$

Therefore, one of the components of microalloyed steel is sulfur, and one goal of this work is to comprehend how alumina inclusions move when there is interfacial tension. The following equation can be used to determine the diffusion coefficient in this situation since sulfur diffuses in steel:

(8) $$D=\frac{kT}{2\pi \mu d}{\left[\frac{m_{1}+m_{2}}{2m_{2}}\right]}^{\frac{1}{2}}$$

where *d* is the diameter of atoms, and *m*_1_ and *m*_2_ are the atomic mass of the solute and solvent, respectively. *T* is the temperature of the melt, *μ* is the viscosity of the molten metal, and *k* is Boltzmann’s constant (1.38 × 10^−23^ J/K).

## 3. Numerical Details

The goal of the current effort was to create a numerical model that can simulate melt flow and forecast inclusion motion behavior in a continuous casting mold. Using a full-scale model of a continuous casting mold, CFD simulation was performed in the first part of the research project. Utilizing Ansys Fluent, a transient two-dimensional numerical model was developed (Academic version: 21.0, ANSYS, Inc., Canonsburg, PA, USA). With the use of the discrete phase modeling technique, inclusion particles (1000 particles at t = 0 s) were then injected. In this study, the aggregation phenomenon of inclusion particles was ignored in favor of the spherical form assumption.

**Figure 2:**
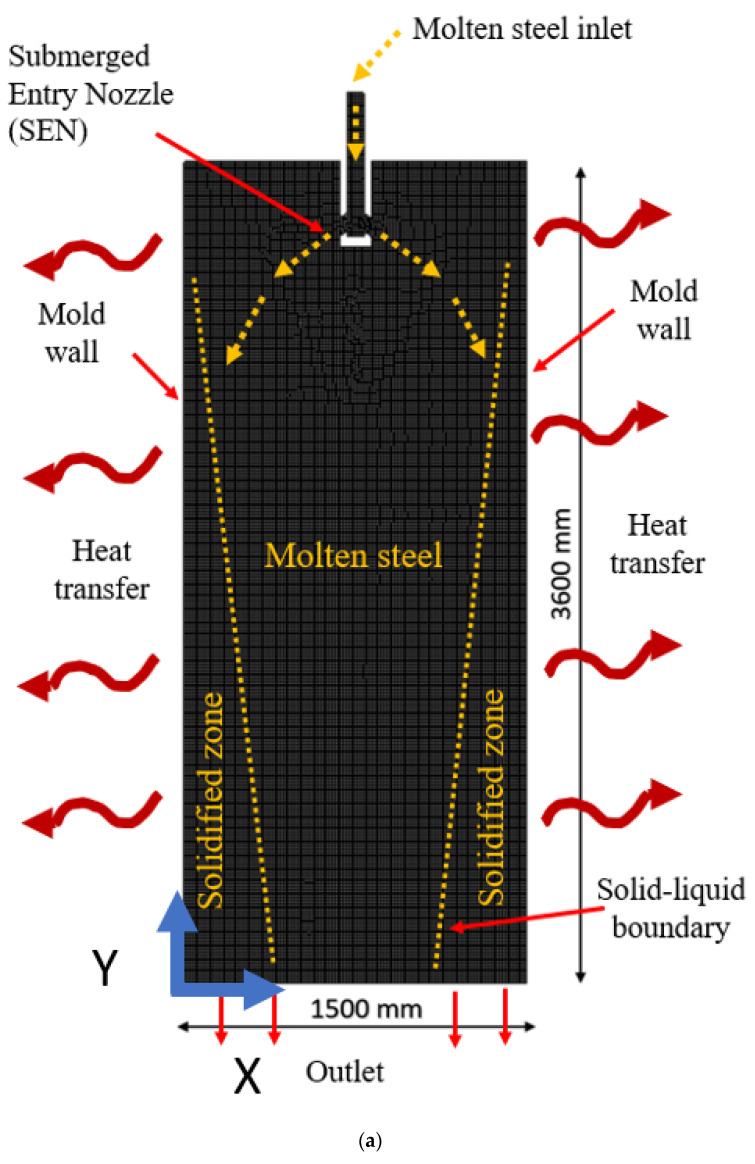

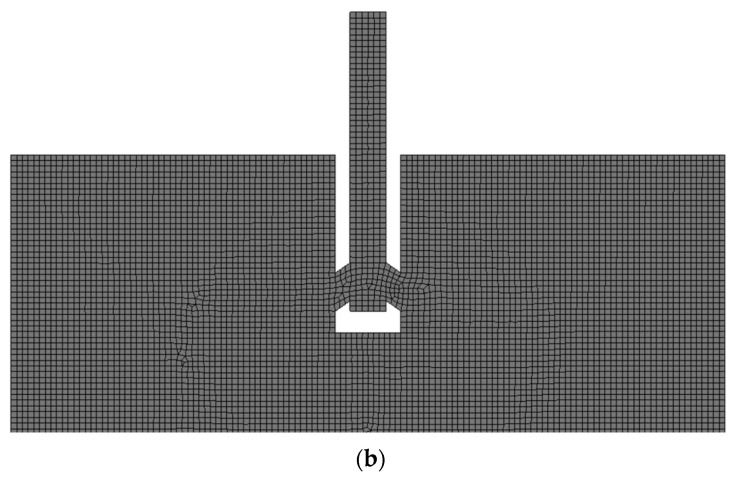
(**a**) Schematic of the domain and (**b**) domain meshing.

A schematic representation of the domain, information on the phases, and the meshed zone are shown in Figure 2a, along with the dimensions of a full-scale mold. The mold’s exterior sidewalls were regarded as stiff walls because of their heat transfer conductivity of 315 W/mk. The simulation’s thermo-mechanical characteristics were derived from earlier work by Siddiqui et al. [28]. The domain’s exterior walls were regarded as convective barriers with appropriate heat transfer rates. We determined the mean velocity along the vertical length of the mold for the grid independence test.

**Figure 3:**
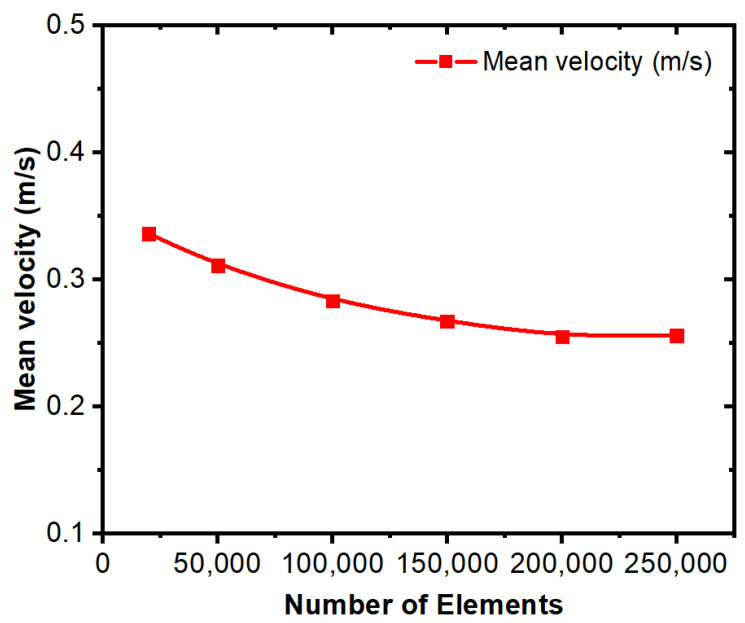
Grid independence test.

The specifics of the grid independence test are shown in Figure 2b (the approximate number of elements is illustrated). It was determined that, in comparison to a greater number of elements, about 200,000 elements produced outcomes that were satisfactory (see Figure 3). According to the grid independence test, the domain’s mid-vertical plane had a mean velocity change of no more than 5%. However, adding more items resulted in a noticeable increase in computing time. By taking an average temperature reading at the mid-vertical plane of the domain, the convergence of the solution was investigated. From Ref. [28], it was possible to determine the thermo-physical characteristics of molten steel and alumina inclusions.

Equation (9) was employed in the simulation to account for the surface tension correlation between molten steel and alumina. Equation (10) was also used in a computer model to measure the interfacial tension between molten steel and alumina inclusions. We used empirical relationships from Jeong et al. [11]. They conducted experiments to determine the surface tension of SPFH590 steel and the interfacial tension of SPFH590 steel and an alumina inclusion. The following formulas can be used to calculate the surface tension of SPFH590 steel:

(9) $$\begin{array}{l} \sigma_{L}=\left(1511+0.08277\;T\right)\\ \;\;\;\;\;\;\;\;\;\;\;\;\;\;\;\;\;\;\;\;\;\;\;\;-\left(1041-0.5156\;T\right)\\ \;\;\;\;\;\;\;\;\;\;\;\;\;\;\;\;\;\;\;\;\;\;\;\;\times \{\ln\left[1+exp\left(-3.583+19846/T\right)\left(wt.\%\;S\right)\right]\} \end{array}$$

where $\sigma_{L}$ is the surface tension, *T* is the temperature in K, and *S* is the sulfur concentration in ppm.

The following equation describes the interfacial stress between SPFH590 steel and an alumina inclusion.

(10) $$\sigma_{PL}=\left\{3050.51+131437.97\times \left(wt.\%\;S\right)-1.544\times 10^{7}{\left(wt.\%\;S\right)}^{2}\;-3.378\times 10^{9}\;{\left(wt.\%\;S\right)}^{3}\right\}\;\;\;\;\;\;\;\;\;\;\;\;+\left\{-0.8498-79.739\times \left(wt.\%\;S\right)+7655.06\times {\left(wt.\%\;S\right)}^{2}\;\;+1.962\times 10^{6}\times {\left(wt.\%\;S\right)}^{3}\right\}T$$

where $\sigma_{PL}$ is the interfacial tension between SPFH590 steel and an alumina inclusion.

**Figure 4:**
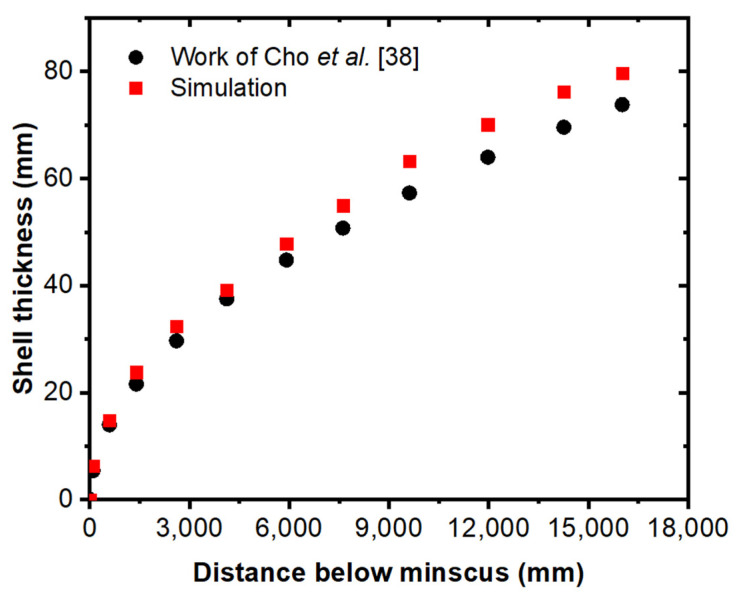
Comparison of solidifying steel shell thickness in the mold (Cho et al. [38]).

Furthermore, sulfur concentration was achieved across the domain by using the species model. In this numerical model, the solidification model was also taken into account. The species continuity equation was solved at each time step during the simulation’s two transient periods. The numerical model for molten steel solidification was validated with the numerical results of Cho et al. [38], as shown in Figure 4. The shell thickness profile was compared with predicted numerical results. The predicted results seem to have good agreement with the previous work of Cho et al. [38].

## 4. Results and Discussion

### Mold and Inclusion Characteristics

In the first part of our work, we investigated the inclusion distribution in a continuous casting mold. A two-dimensional discrete phase model (DPM) was used to analyze and track the movement of inclusion particles. In addition to this solidification, a model was employed to simulate the solidified zone in the mold. A suitable turbulence model, the k-ɛ realizable model, was selected to properly configure the turbulence flow of molten steel. Further, inclusions were assumed to be spherical in shape and non-conglomerating to simplify the numerical modeling procedure.

**Figure 5:**
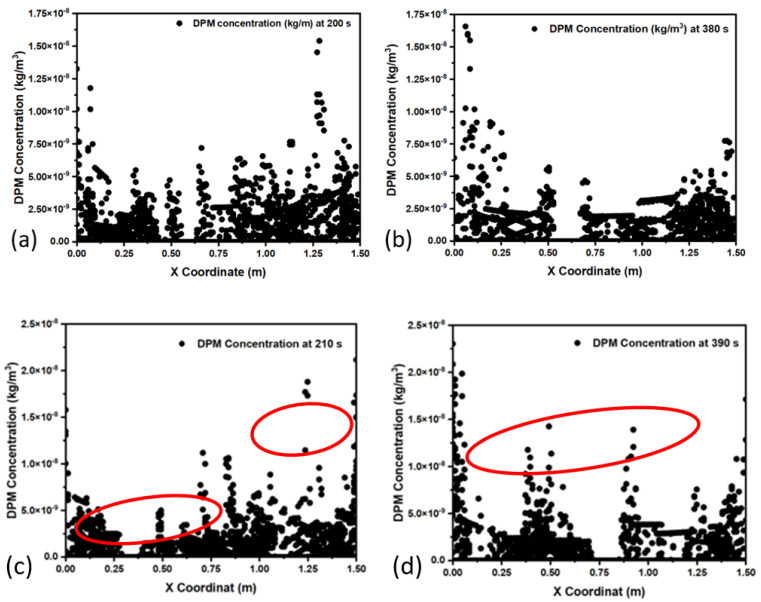
Inclusion (DPM) concentration in the mold at 1853 K; sizes: (**a**,**b**) 5-micron diameter and (**c**,**d**) 100-micron diameter.

Figure 5a,b shows the distribution of 5-micron-diameter inclusions at the mid-section of the mold at different periods. It can be observed that inclusion concentrations (calculated by the discrete phase model, DPM) increase over time at a given section. Secondly, it is also observed that a major part of the inclusion distribution is available on the left and right sides of the mold, which are solidifying boundary layers. Further, the midzone of the mold has a lower concentration of inclusions. The inclusion motions are affected by several parameters, such as the velocity of the local zone, buoyancy force, natural convection, and interfacial tension. It is expected that inclusions move to the solidifying zone due to an increase in the thermal gradient and a lower local velocity field.

**Figure 6:**
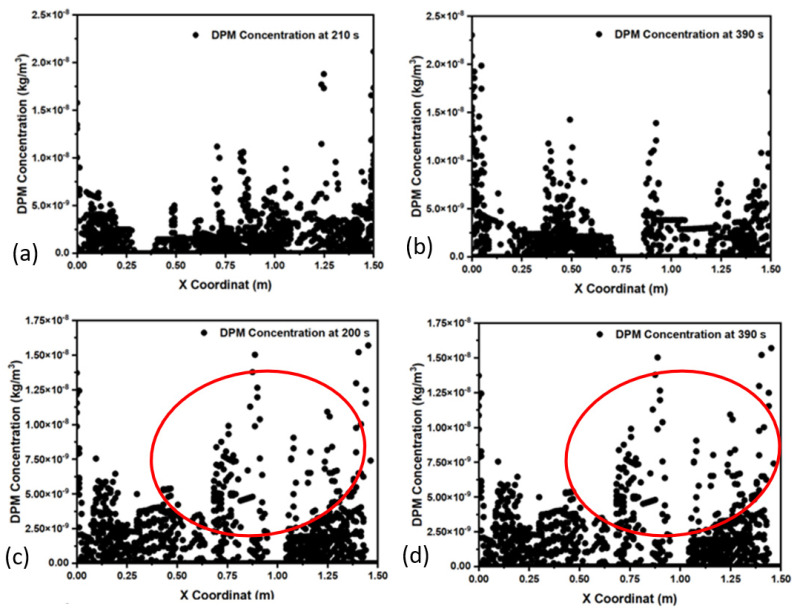
Inclusion (DPM) concentration with a 100-micron diameter in the mold at (**a**,**b**) 1853 K and (**c**,**d**) 1893 K.

Figure 5c,d show the distribution of 100-micron-diameter inclusions at the mid-section of the mold at different periods. In this case, inclusion concentrations have different distribution characteristics as compared with small (5-micron size) inclusions. It can be noticed here that inclusions have a lump-sum uniform distribution over the *x*-axis length. However, it cannot be denied that some part of the inclusion population is concentrated towards the solidifying boundary. A significant portion of the inclusion distribution is noticed on the left and right sides of the mold, which are hardening border layers. We later study the larger-sized inclusion particles and their distribution in the mold zone. It was found that inclusions are more evenly concentrated in the mold zone as compared to the smaller sizes. This implies that larger inclusions can be more influenced by melt flow velocity and buoyancy forces. Furthermore, the effect of temperature on the inclusion distribution was investigated to analyze the impact of natural convection and interfacial tension. Figure 6 shows the inclusion concentration on the same plane but with elevated temperature conditions. It can be seen that the inclusion concentration is more evenly distributed in the mold as compared to lower-temperature conditions.

**Figure 7:**
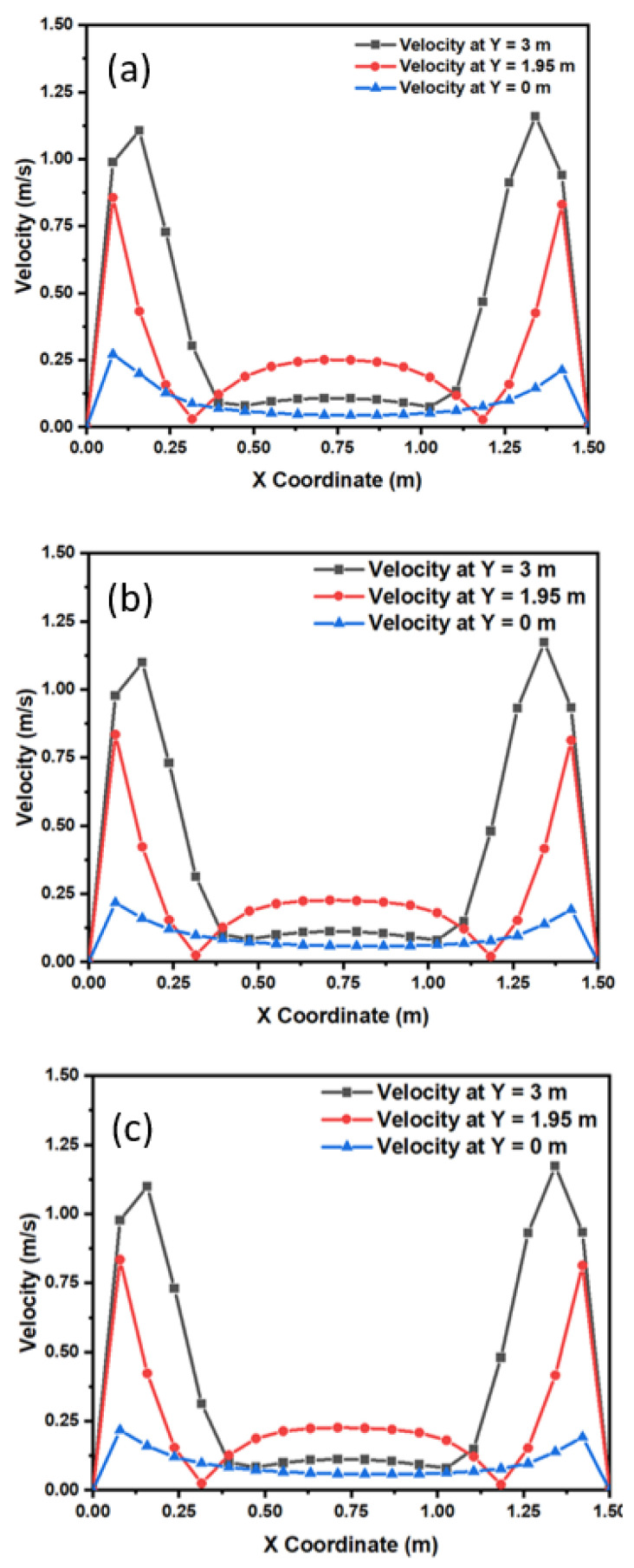
The inclusion velocity profile in the mold at 1853 K; size: (**a**) 5 microns, (**b**) 100 microns, and (**c**) 300 microns.

Figure 7 shows the velocity profiles of 5-, 100-, and 300-micron inclusion particles at different sections of the mold. On the top side of the mold, we can see that inclusions have a higher flow velocity especially at the left and right sides of the solidifying boundary. Similarly, for the midzone, we can see that the velocity of inclusions has been reduced due to a reduction in flow field velocity. The flow field velocity is reduced due to a reduction in temperature and an increase in the viscosity of the melt. In the lower zone (Y = 0 m), we can observe the minimal velocities of inclusion particles. The velocity profile of 100-micron inclusion particles at different sections of the mold is shown in Figure 7. We can see that inclusions have a larger flow velocity on the top side of the mold, especially on the left and right sides of the solidifying border. Similarly, we can see that the velocity of inclusions decreases at the midzone (Y = 1.95 m) due to a decrease in flow field velocity. Because of the lower temperature and higher viscosity of the melt, the flow field velocity is lower. We can see the lowest velocities of inclusion particles in the bottom zone (Y = 0 m).

Further, it depicts the velocity curve of 300-micron inclusion particles at various mold sections. On the top side of the mold, especially on the left and right sides of the solidifying border, inclusions have a higher flow velocity. Similarly, due to a drop in flow field velocity, the velocity of inclusions is reduced at the midzone (Y = 1.95 m). The flow field velocity is reduced because of the lower temperature and increased viscosity of the melt. The bottom zone (Y = 0 m) has the slowest inclusion particle velocities.

**Figure 8:**
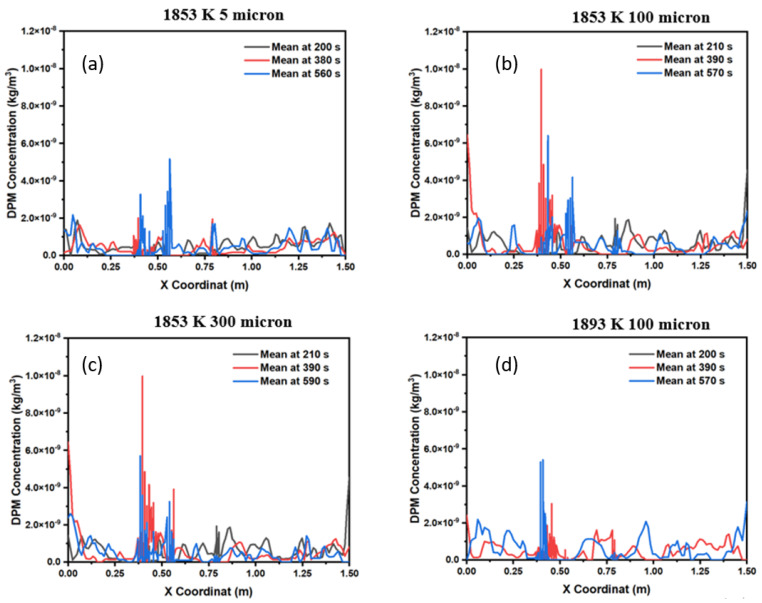
Inclusion concentration profile in the mold: (**a**) 5 microns, (**b**) 100 microns, (**c**) 300 microns, and (**d**) 100 microns (at 1893 K).

The average inclusion concentration profile on the *x*-axis of the domain is shown in Figure 8 with respect to inclusion size and time. Figure 8a represents the distribution of small inclusions (5-micron diameter) over different timescales, namely, 200 s, 380 s, and 560 s. It is observed that the average inclusion concentration remains unvaryingly distributed throughout the domain. However, during a certain period of the simulation, two peaks can be observed in the domain. This suggests that the inclusion distribution increases near the middle zone of the domain. Further, when we changed the inclusion size in the same conditions, it was observed that inclusions are concentrated in less time (390 s), as shown in Figure 8b. A similar pattern is also observed in Figure 8c, where the inclusion diameter is significantly increased to 300 microns. In another case, we tried to see the effect of the inlet temperature on the average distribution of inclusions under the same conditions. Figure 8d shows the effect of the inlet temperature change. It was observed that the peak pattern of the average distribution has an impact on temperature, and inclusion concentrations are formed on a larger timescale.

**Figure 9:**
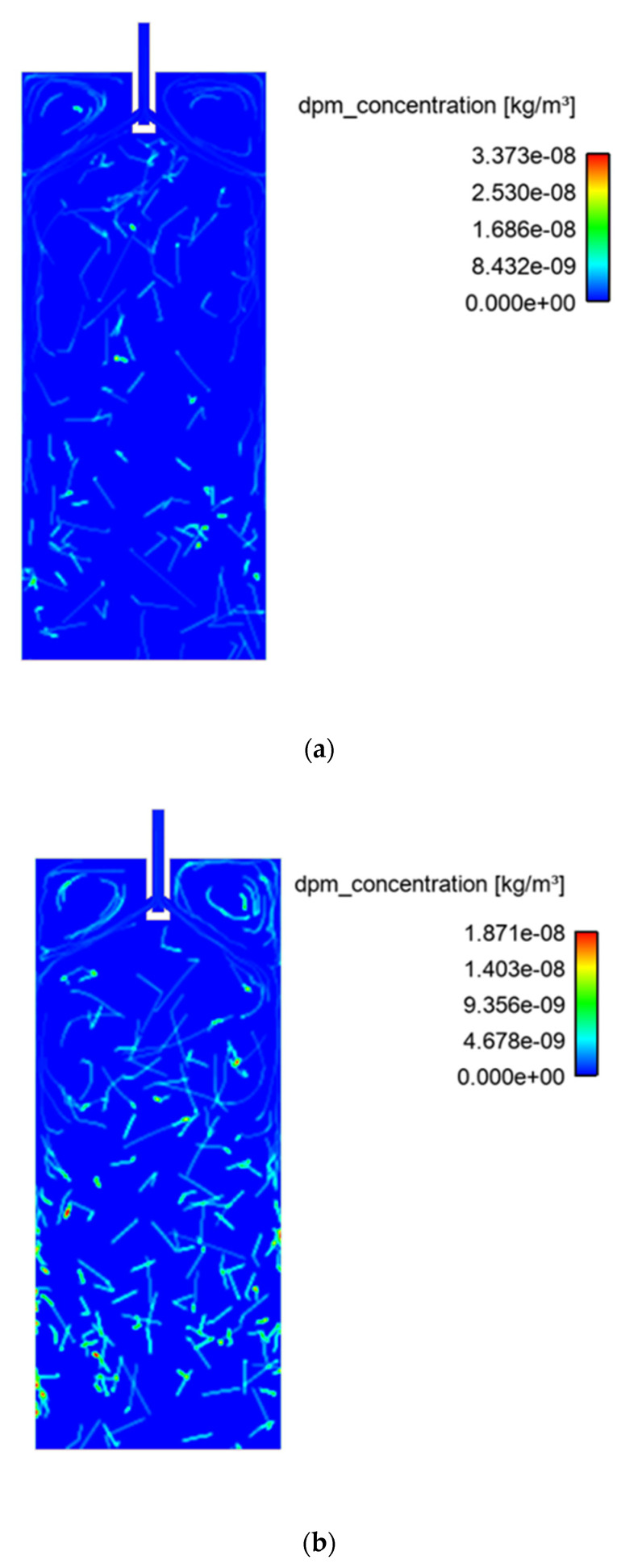

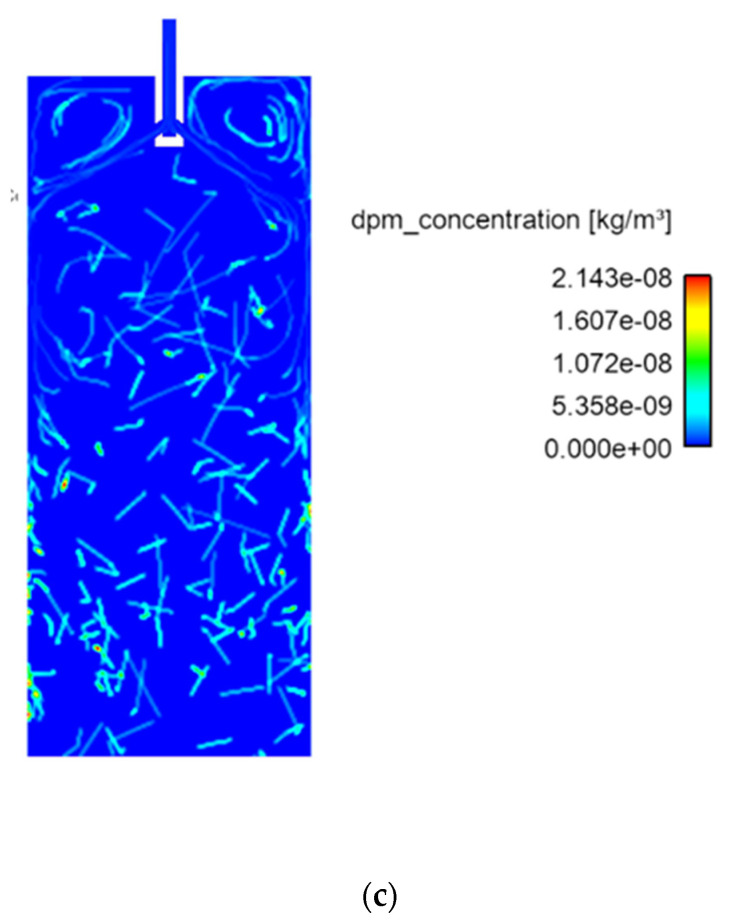
Inclusion concentration profile in the mold at 1853 K; diameter size: (**a**) 5 microns, (**b**) 100 microns, and (**c**) 300 microns.

**Figure 10:**
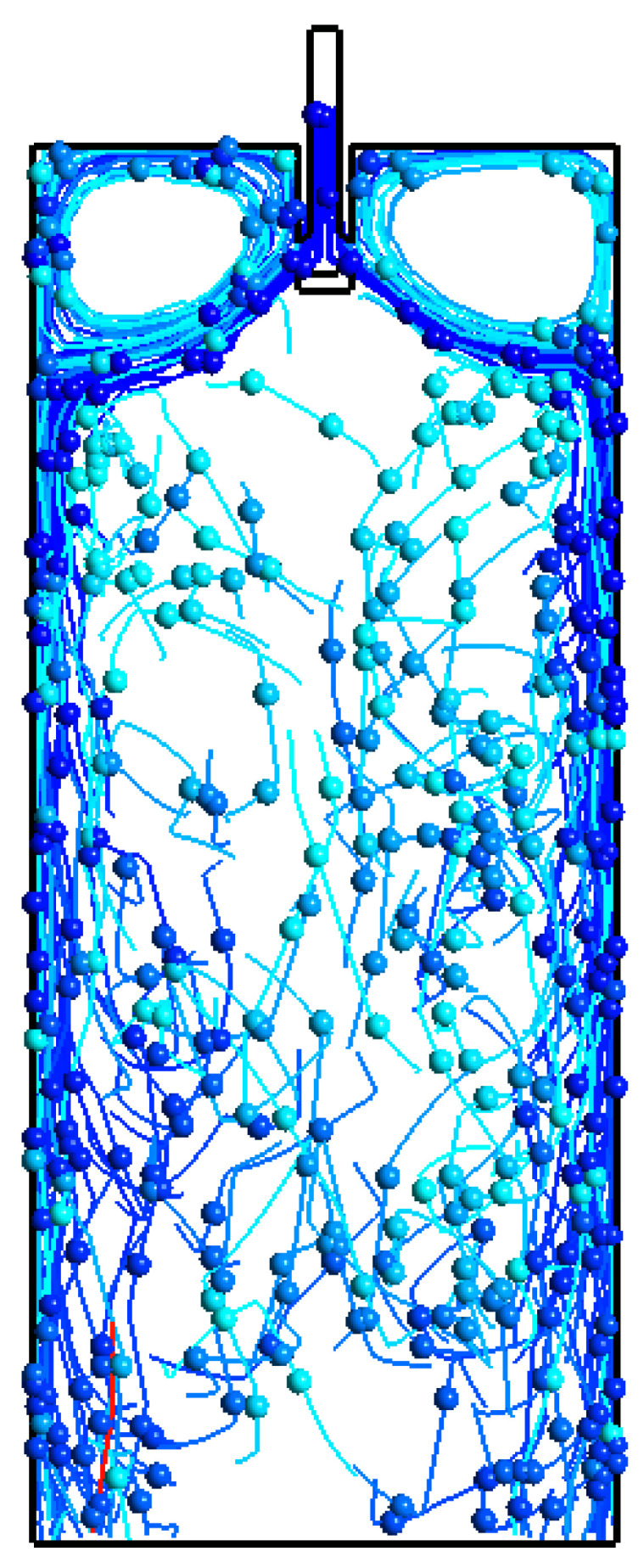
Alumina inclusion motion in the mold.

In Figure 9, the inclusion concentration profile is shown in the whole domain of the simulation by using different sizes of inclusions. It can be noted that the inclusion distribution is mostly on the middle-left side of the mold. On the other hand, we can see almost the same level of inclusion distribution. In addition to this, inclusions more or less have the same concentration at six different time lengths. The inclusion distribution density is depicted in Figure 9a–c throughout the domain for different cases. This density concentration further confirms the inclusion aggregation towards the solidifying boundary zone. Further, the inclusion motion with streamlines is shown in Figure 10. The position and motions of inclusions are shown at a well-established flow time (1000 s) in the mold.

**Figure 11:**
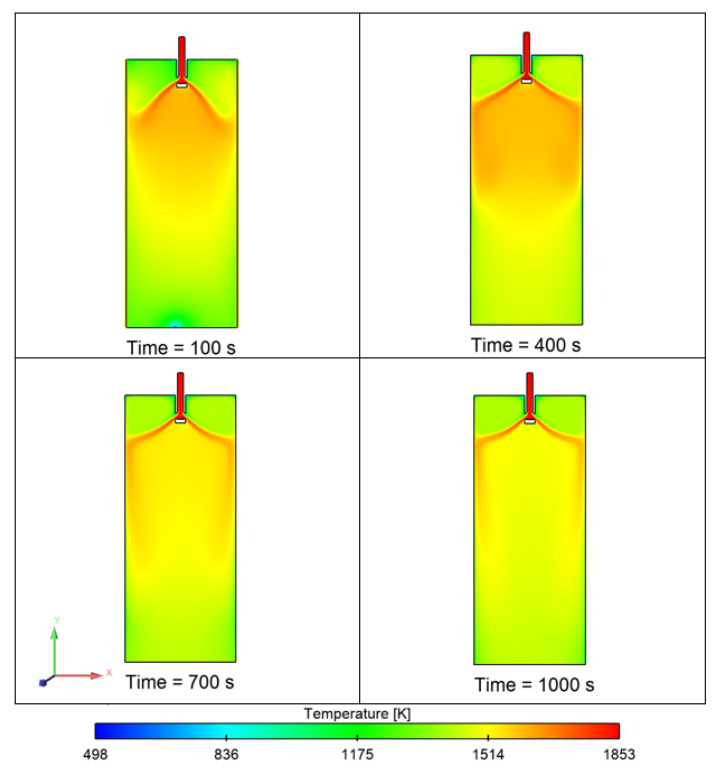
Temperature contour of the mold domain (initial melt temperature: 1853 K).

**Figure 12:**
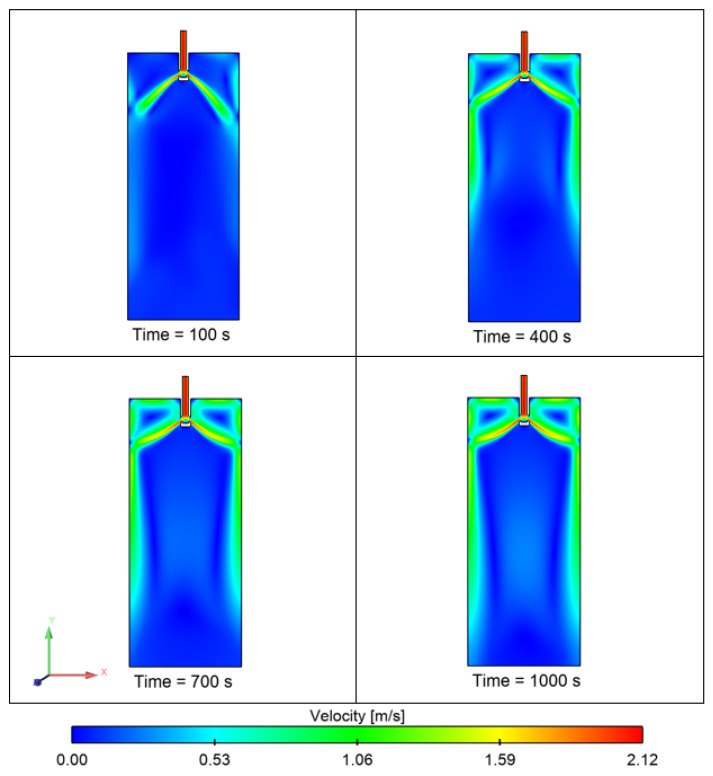
Velocity contour of the mold domain (initial melt temperature: 1853 K).

**Figure 13:**
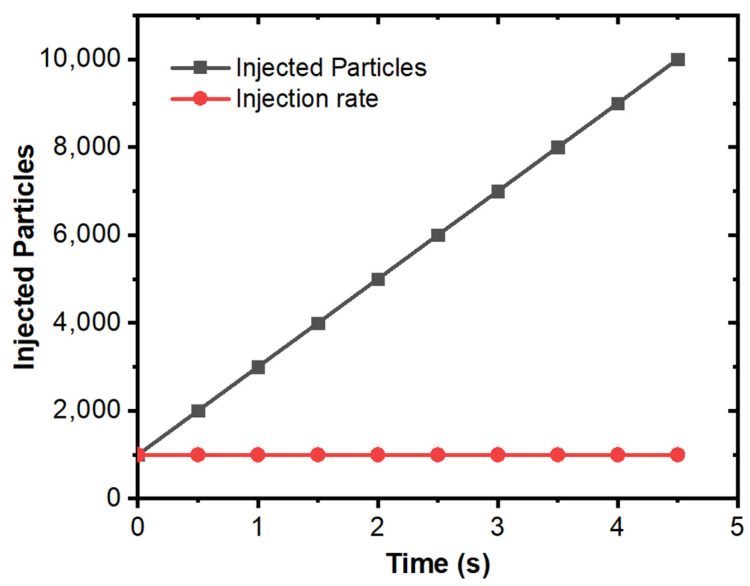
Injection rate and total particles in the mold using DPM modeling.

**Figure 14:**
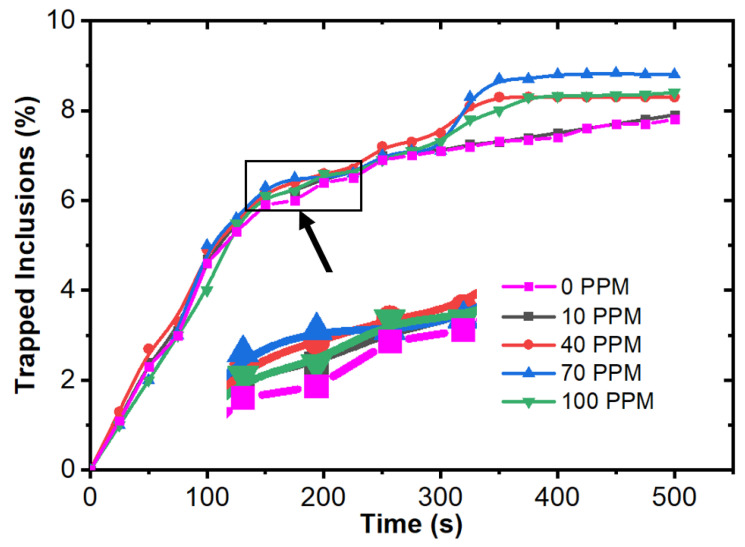
Trapped inclusions near the solid–liquid boundary in the mold at different sulfur concentrations.

Figure 11 shows the temperature contour of the domain at different time intervals. The initial temperature of the melt inflow was 1853 K. The temperature of molten steel decreases after some time, and the boundary layer of solidifying steel is visible in the domain. Figure 12 shows the velocity contour in the domain at different time intervals. Figure 13 shows the total number of inclusion particles available in the domain as a function of injection time using the discrete phase model. We studied the effect of sulfur concentration on the entrapment of alumina inclusions near the solid–liquid boundary layer in the mold. The sulfur concentration affects the interfacial tension at the solid–liquid boundary, and thus, it impacts the motion of alumina inclusions due to interfacial tension. Figure 14 shows the trapped inclusions near the solid–liquid boundary in the mold at different sulfur concentrations. It can be noted here that when there was no mixing of sulfur, the entrapment percentage was low with respect to time. Because of that condition, we did not utilize the empirical relationship in the simulation. However, the entrapment percentage varied in the case of sulfur mixing (using the empirical relationship in modeling). It can be noticed that a 10 ppm sulfur concentration does not have much impact. However, as we increase the sulfur concentration up to 70 ppm, we can see that there is a significant rise in the entrapment of alumina inclusions in the solidifying boundary layer. Another important outcome that we notice here is that the maximum entrapment of alumina inclusions occurs at 70 ppm, but the entrapment percentage decreases at 100 ppm sulfur. The variation in entrapment can be directly correlated with the experimental correlation between the interfacial tension and sulfur concentration, as stated in Equations (9) and (10).

**Figure 15:**
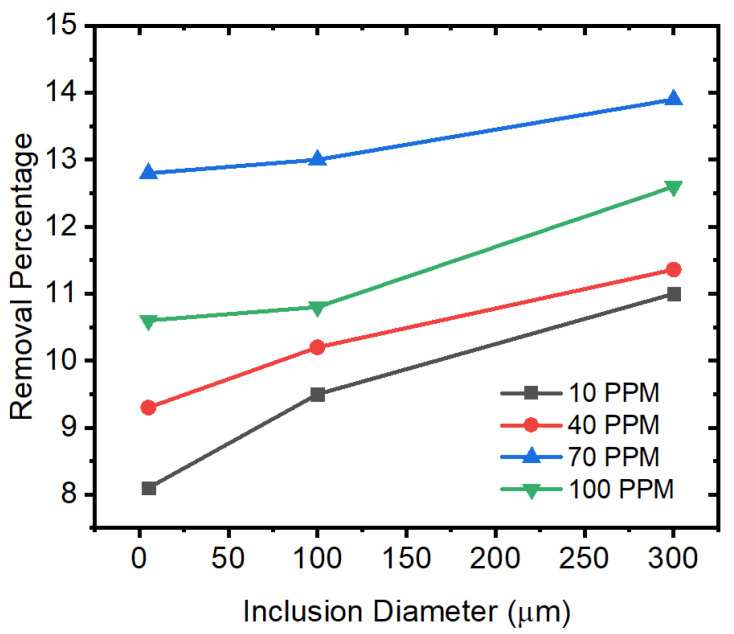
Removal percentage of alumina inclusions in different conditions of sulfur concentration and inclusion diameters.

Figure 15 shows the removal percentage of inclusions from the outlet (i.e., outlet inclusion particles are assumed to have moved out or absorbed in the solidified shell). It can be noted here that the removal percentage varies according to the sulfur concentration (around 4%). We can observe that an increase in sulfur ppm up to 70 ppm leads to higher removal percentages, but the results are the opposite at 100 ppm. Moreover, larger particles are more prone to move out of solidified shells as compared to smaller particles.

**Figure 16:**
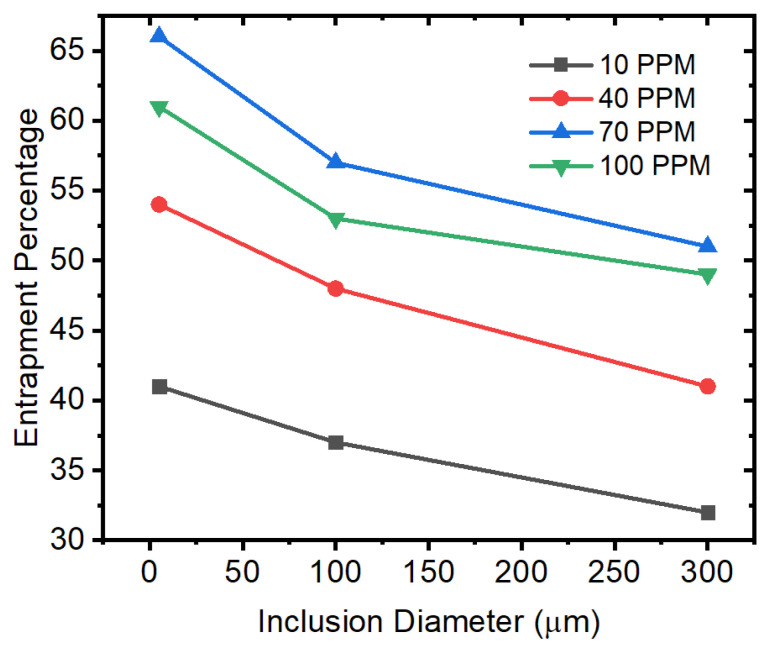
Entrapment (at the top surface) of alumina inclusions under different conditions of sulfur concentration and inclusion diameters.

We also studied the entrapment of inclusion particles at the top surface of the mold. In a typical continuous casting mold, the slag layer is artificially created on the top surface for many reasons. One of the advantages is that inclusion particles become stuck in the slag layer. Thus, in this simulation model, we considered the entrapment of particles when they reach the top surface. Hence, a particular inclusion particle’s velocity is frozen at zero, and the discrete phase model stops tracking it. Figure 16 shows the entrapment of inclusion particles on the top surface of the mold. We observed that the smaller-sized inclusion particles have higher chances of entrapment (more than 25%) and are thus removed from the molten metal. However, the larger-sized inclusion particles sustain their motion in the melt for a longer period, and their percentage entrapment is lower. The other conclusion that we made is that interfacial tension plays an important role in entrapment, as can be seen in Figure 16. An increase in sulfur concentration up to 70 ppm leads to more entrapment; however, an increase to 100 ppm does not make a more significant difference.

**Figure 17:**
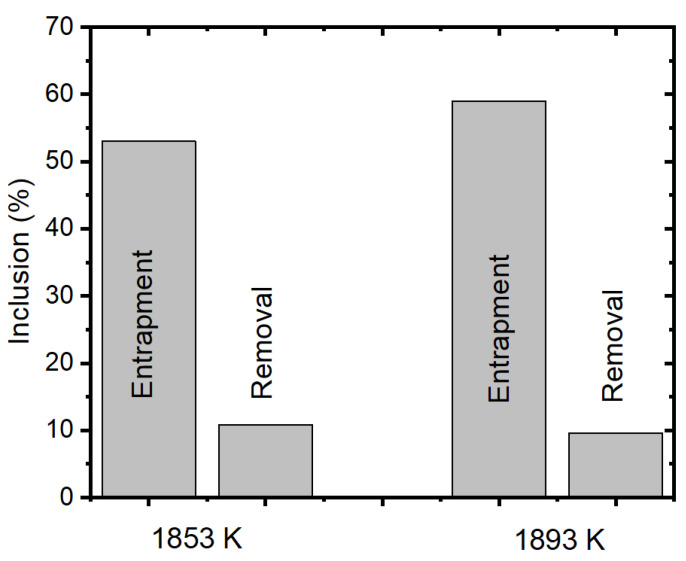
Entrapment and removal (from outlet) percentages of alumina inclusion.

Further, we also studied the effect of melt inlet temperature and its effect on inclusion entrapment and removal (from the outlet/solidified shell). Figure 17 shows the entrapment and removal (from the outlet) percentage of alumina inclusions. It can be noted that the inclusion entrapment percentage is increased as the inlet temperature is increased. The temperature at the inlet and top surface zone affects the surface tension and melt flow characteristics. Secondly, we can also observe that the removal percentage does not vary significantly.

## 5. Conclusions

A numerical model was developed to study alumina inclusion motion behavior in a continuous casting mold. Initially, we investigated the inclusion motion behavior in a continuous casting mold using a two-dimensional CFD model. The inclusion motion characteristic was studied with different parameters, such as the input temperature of molten steel in the mold and the size of the inclusion particles. Secondly, we focused on inclusion motion under the influence of interfacial tension. The effect of surfactant concentration (sulfur in this work) on the motion of alumina inclusions was studied. The following are the main conclusions obtained from this research work:

- A transient, two-dimensional model of a continuous casting mold was developed using Ansys Fluent software. Alumina inclusions in microalloyed steel were tracked under the condition of solidification at the boundary side. Further, the empirical relationship of interfacial tension was used to study the effects of temperature and surfactant concentration (sulfur) on inclusion motion near the solidifying boundary layer in the mold.
- The simulation findings indicate that the size of the inclusions and the inlet temperature have significant effects on the distribution of inclusions. It was also observed that a major part of the inclusion distribution was on the left and right sides of the mold, which are solidifying boundary layers. Further, the midzone of the mold had a lower concentration of inclusions. The inclusion motions were affected by several parameters, such as the velocity of the local zone, buoyancy force, natural convection, and interfacial tension.
- The inclusion diameter affected the inclusion distribution throughout the domain. Larger-sized inclusions had different distribution characteristics as compared to small (5-micron size) inclusions. It was found that larger inclusions were more evenly concentrated in the mold zone as compared to the smaller sizes. This implies that larger inclusions can be more influenced by the melt flow velocity and buoyancy forces.
- The effect of temperature on the inclusion distribution was investigated to analyze the impact of natural convection and interfacial tension. It was noticed that the inclusion concentration was more evenly distributed in the mold as compared to lower-temperature conditions.
- The entrapment percentage of alumina inclusions was low over time when the interfacial tension equation was not used. However, the entrapment percentage varied in the case of sulfur mixing (using the empirical relationship in modeling). It was found that a 10 ppm sulfur concentration did not have much impact. However, we observed that there was a significant rise in the entrapment of alumina inclusions in the solidifying boundary layer at sulfur concentrations up to 70 ppm. Another important outcome was that the maximum entrapment of alumina inclusions occurred at 70 ppm, but the entrapment percentage was lower at 100 ppm sulfur.
- It was found that the removal percentage varied according to the sulfur concentration. The addition of sulfur at concentrations from 10 ppm to 70 ppm resulted in around a 4% increase in the removal of alumina inclusions (trapped in the solidifying shell), except for the 100-ppm case.
- The smaller-sized inclusion particles had a 25% higher chance of entrapment at the top level of the mold (slag layer). However, the larger-sized inclusion particles sustained their motion in the melt for a longer period, and their percentage entrapment was lower.
- It can be noted that the inclusion entrapment percentage increased as the inlet temperature was increased. The temperature at the inlet and top surface zone affected the surface tension and melt flow characteristics. Secondly, we also observed that the removal percentage did not vary significantly.

## Figures and Tables

**Table 1:** Chemical composition of selected steel (SPFH590) (wt%).

Element	C	Mn	Si	P	Al	Nb
Concentration	≤0.1	≤3.0	≤0.5	≤0.1	≤0.1	≤0.1

**Table 2:** Thermo-physical properties of SPFH590 steel.

Parameters	Values
Density of molten steel [36]	ρ (kg m^−3^) = 8621.17 − 0.88T
* Viscosity of molten steel [36]	μ (mPa s) = 0.2388 ∗ exp(47.44/(RT))
Specific heat [37]	750 J kg^−1^ K^−1^
Thermal conductivity [37]	41 W m^−1^ K^−1^
Surface tension (σL) and interfacial tension (σPL)	Equations (9) and (10) {Ref: [10,11]}
Solidus temperature	1781 K
Liquidus temperature	1798 K
Alumina inclusion size	5, 100, and 300 μm

* where μ is the viscosity, R is the molar gas constant, and T is the absolute temperature (K).

**Table 3:** Process Parameters.

Process Parameters	Values
Mold width	1500 mm
Mold length	3600 mm
SEN submergence depth	160 mm
Nozzle port downward angle	15 degree
Inlet velocity	2 m/s
Outlet	Pressure outlet condition
Alumina inclusion density	2500 kg/m^3^
Shell surface temperature	1273 K
Mold conductivity	315 W/mk
Latent heat	272,000 (J/kg)

## Data Availability

Available on request.
